# Restoring the Oxidase-Like Activity of His@AuNCs for the Determination of Alkaline Phosphatase

**DOI:** 10.3390/bios11060174

**Published:** 2021-05-30

**Authors:** Fanfan Xiao, Yuting Yu, Yang Wu, Lili Tian, Guoyan Zhao, Hailong Pang, Jie Du

**Affiliations:** 1 College of Life Sciences, Northwest Normal University, Lanzhou 730070, China; 2018212256@nwnu.edu.cn (F.X.); 2017212141@nwnu.edu.cn (Y.Y.); 2020212651@nwnu.edu.cn (Y.W.); 2018212262@nwnu.edu.cn (L.T.); 2018212254@nwnu.edu.cn (G.Z.); Phl-3088@163.com (H.P.); 2 Key Laboratory of Bioelectrochemistry & Environmental Analysis of Gansu Province, Northwest Normal University, Lanzhou 730070, China

**Keywords:** His@AuNCs/GO, oxidase-like activity, PPi, ALP, colorimetric detection

## Abstract

In this paper, we propose a simple colorimetric method for the sensitive and selective detection of alkaline phosphatase (ALP) activity based on the turn off/turn on oxidase mimic activity of His@AuNCs. His@AuNCs/graphene oxide hybrids (His@AuNCs/GO) were easily obtained using the self-assembly method with poly (diallyldimethylammonium chloride) (PDDA)-coated GO and showed high oxidase-like activity compared with His@AuNCs. We found that the pyrophosphate ion (P_2_O_7_^4−^, PPi) could effectively inhibit the oxidase mimic activity of His@AuNCs/GO, and the hydrolysis of PPi by ALP restored the inhibited activity of His@AuNCs/GO, enabling them to efficiently catalyze the oxidation of 3,3′,5,5′-tetramethylbenzidine (TMB) to generate the blue oxidized product oxTMB. The intensity of the color showed a linear dependency with the ALP activity. ALP was detected in the linear range of 0–40 mU/mL with a low detection limit (LOD) of 0.26 mU/mL (S/N = 3). The proposed method is fast, easy, and can be applied to monitor the ALP activity in serum samples accurately and effectively, which suggests its practicability and reliability in the detection of ALP activity in clinical practice.

## 1. Introduction

Alkaline phosphatase (ALP) is widely present in the tissues and organs of biological organisms (e.g., bones, liver, intestine, kidneys, and placenta) and can catalyze the hydrolysis and transphosphorylation of a variety of phosphate compounds, including DNA, proteins, and other small molecules [1,2]. Several diseases, such as bone cancer [3], prostate cancer [4], and diabetes [5] and liver diseases [6], are influenced by ALP activity, and thus it has been recognized as a significant biomarker in medical diagnostics.

ALP is frequently utilized as an enzyme label in biochemical assays, such as the enzyme-linked immunosorbent assay (ELISA) [7]. Therefore, simple, sensitive, and reliable assays are highly desirable in the detection of ALP activity in the research and diagnosis of diseases [8,9,10]. Several conventional methods are typically used for ALP activity determination, such as electrochemistry [11,12], fluorometry [13,14], colorimetry [15,16], etc.

Among these, the colorimetric method has spurred great interest owing to its intrinsic simplicity, speed, and cost-effectiveness. In the standard method that is often employed, colorless *p*-nitrophenyl phosphate (pNPP) is used as the substrate and is converted into the yellow *p*-nitrophenolate using ALP as the catalyst, and the reaction results in realistic colorimetric assays [17]. However, previous colorimetric methods often showed inferior sensitivity and reproducibility, as direct measurement of the subtle color change of the substrates or products of ALP was extremely difficult [18]. To address this shortfall, the cascading of an additional amplification process to establish the sensitivity of ALP is of value. 

Research studies have reported that using the substrates or products of ALP to regulate plasmonic gold and silver nanomaterials [19,20,21] helped to achieve signal amplification. For example, using plasmonic gold nanorods, Gao et al. designed a colorimetric assay to determine the ALP activity. In this method, ALP was used as the catalyst in the hydrolysis of ascorbic acid-2-phosphate (AA-P) to ascorbic acid (AA); the AA was then used to reduce the silver ions to generate a silver shell on the Au nanoclusters (AuNCs), which led to a perceptible color change producing the signal readout [14]. 

Although these approaches can provide a simple and visual detection of ALP, plasmonic nanomaterials suffer from non-specificity of ions in complex biological samples [22], resulting in poor sensitivity and a limited linear range. Recently, alternative strategies, using the substrates and products of ALP, have been developed to modify nanozymes (nanomaterials with intrinsic enzyme-like properties), which could result in signal amplification [23,24]. 

For example, Tian et al. reported an ALP assay employing MnO_2_ nanosheets as the signal amplifier [25]. The MnO_2_ nanosheets catalyze the oxidation of the colorless substrate TMB to form the blue oxidized product (oxTMB) and ALP catalyzes the hydrolysis of AA-P to AA, which could weaken the activity of the MnO_2_ nanosheets, subsequently weakening the MnO_2_-controlled cascade signal. As a result, the readout signal amplification corresponding to the ALP activity can be obtained from the change in absorption. Chen et al. also developed a nanozyme-based signal amplification method by modifying the catalytic activities of gold nanoclusters (AuNCs) for the colorimetric detection of ALP activity [26]. 

These nanozyme-based ALP activity assays can achieve signal amplification [27,28,29]. Some methods can even be efficiently used in colorimetric immunoassays. However, the sensitivities of these methods are highly dependent on the enzyme-like catalytic activity of the nanozymes. Usually, the catalytic activities of nanozymes are low when compared with those of natural enzymes, which limits their application. Therefore, the sensitivity of nanozyme-based ALP assays must be enhanced by improving the catalytic activities of the nanozymes. 

Anchoring NPs on suitable nanoscale supporters to form hybrid nanomaterials has recently been found to be an effective method to enhance the catalytic activity of nanozymes [30,31,32,33,34]. Studies have reported that the enzyme-like activity of metal NCs can be greatly enhanced by decorating them on two-dimensional nanomaterials, such as graphene and graphitic carbon nitride. For instance, Wu et al. demonstrated that AuNPs grown in situ on g-C_3_N_4_ nanosheets are hybrid nanozymes, have higher catalytic activities than both C_3_N_4_ nanosheets and AuNPs, and can be applied in the colorimetric detection of glucose [35]. Chen et al. constructed a colorimetric hydrogen peroxide (H_2_O_2_) sensor using TMB in an acetate buffer (pH 4) solution, based on Fe/Pt nanoparticles decorated on graphene oxide (GO) nanosheets as oxidase mimics [36]. In our recent work, we improved the catalytic activity of histidine-protected AuNCs (His@AuNCs) by decorating the nanoclusters on reduced graphene oxide (RGO), with the nitrite modulating the oxidase mimic activity of the His@AuNCs, thus, leading to the development of a nitrite sensor [37].

**Scheme 1:**
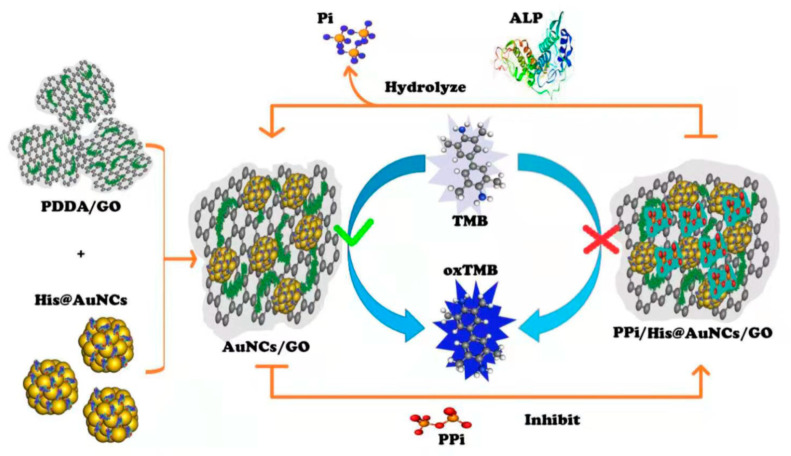
Illustration of the colorimetric sensing strategy for ALP activity based on PPi-mediated catalytic activity of His@AuNCs/GO.

In this work, a novel nanozyme-based colorimetric platform for the simple, sensitive, and selective screening of ALP activity was successfully developed. As shown in Scheme 1, His@AuNCs can be efficiently decorated on poly (diallyldimethylammonium chloride) (PDDA)-functionalized GO using a facile sequential process, and the resulting His@AuNCs/GO with improved oxidase-like performance can be used to effectively catalyze TMB to oxTMB. Interestingly, the catalytic activity of His@AuNCs/GO dramatically decreased with the presence of pyrophosphate ion (PPi) in the buffer solution. 

In the presence of ALP, PPi is hydrolyzed to phosphate (Pi), resulting in the restoration of the catalytic activity of His@AuNCs/GO, enabling the detection of the ALP activity. The proposed method shows a good linear relationship in the range of 0–40 mU/mL with a LOD of 0.26 mU/mL. In addition, ALP in human serum samples was detected successfully using this method, which indicates its potential as a promising candidate in biological and biomedical applications.

## 2. Materials and Methods

### 2.1. Chemicals and Instruments

L-Histidine (His), ALP, and TMB were purchased from Aladdin Chemistry Co., Ltd. GO was purchased from Nanjing XFNano Mstar Technology. Ltd. (Nanjing, China). Sodium pyrophosphate tetrabasic decahydrate (Na_4_P_2_O_7_·10H_2_O) and PDDA (MW = 400,000–500,000, 20 wt% in water) were purchased from Sigma-Aldrich (Shanghai, China). Chloroauric acid tetrahydrate (HAuCl_4_), poly(N-vinyl-2-pyrrolidone) (PVP) (K30, MW = 30,000–40,000), KCl, and other chemicals were purchased from Sinopharm Chemical Reagent Co. Ltd. (Shanghai, China). Dialysis bags (molecular weight cut-off (MWCO) of 1000 Da; regenerated cellulose (RC) membranes) were purchased from Shanghai Yuanye Bio-Technology Co., Ltd. (Shanghai, China). All chemicals were of analytical grade and were directly used without further purification. All solutions were prepared with deionized water (18.25 MΩ cm).

We used transmission electron microscopy (TEM) by a TECNAI G^2^ field emission projection electron microscope (FEI TECNAI G20, Waltham, MA, USA). We used a Nicolet iS50 Fourier transform infrared spectroscope (Thermo Scientific, Waltham, MA, USA) for infrared spectra collection. We obtained all absorption spectra on a UV-1800 ultraviolet–visible spectrophotometer (Shimadzu, Japan). We used a pH meter (Sartorius AG, Germany) for pH adjustment.

### 2.2. Synthesis of His@AuNCs

His@AuNCs were prepared based on a previous work by our group [37]. In this method, an aqueous solution of HAuCl_4_ (1 mL, 10 mM) was blended with an aqueous solution of histidine (3 mL, 0.15 mM) and the mixture was incubated for 2 h at 25 °C. The color of the mixture changed from light yellow to light brown; when the mixture was irradiated with 365 nm ultraviolet light, the mixture emitted a strong blue light. The His@AuNCs solution that was obtained was isolated from the precipitates by centrifugation at 14,000 rpm for 15 min and then washed using a dialysis bag (MWCO: 1000 Da) until the pH of the solution became neutral. The His@AuNCs obtained were concentrated by freezing and drying under vacuum, were re-dispersed in an aqueous solution, and were stored at 4 °C until further use.

### 2.3. Synthesis of His@AuNCs/GO

First, PDDA/GO was prepared according to the literature, with a slight modification [38]. In this method, 40 mg PVP was added to 10 mL GO solution (0.25 mg/mL) and sonicated for 30 min. The resulting black solution was washed, centrifuged three times, and dissolved in 2.5 mL of water. Afterwards, 0.05 mL of 20 wt% PDDA was mixed well with 8.4 mL of 0.625 M KCl, followed by the injection of 2.1 mL PVP-capped GO; the resulting solution was sonicated for 1.5 h. 

The black sediment was washed with deionized water and subjected to centrifugal separation. Finally, the products were re-dispersed in 2 mL water and named as PDDA/GO. For the fabrication of His@AuNCs/GO, 1 mL PDDA/GO (1 mg/mL) was added to 40 mL of the His@AuNCs solution under stirring (Figure S1). The solution was then sonicated for 3 min before being left to stand overnight, then washed three times, and dissolved in 2 mL water (final concentration: 0.5 mg/mL). This His@AuNCs/GO solution (0.005 mg/mL, Figure S2) was used for the subsequent experiments

### 2.4. Oxidase-Like Activity of His@AuNCs/GO

The oxidase-like activity of His@AuNCs/GO was tested using TMB as the substrate. Similarly, we used His@AuNCs instead of His@AuNCs/GO to conduct control experiments under the same conditions. For the subsequent experiments, 10 μL His@AuNCs/GO (0.5 mg/mL) was added to 1000 μL of the total reaction solution. Based on the maximum concentration of oxTMB, the other experimental parameters were optimized as follows (Figure S3): (1) the pH and concentration of acetate buffer were maintained at 3.2 and 50 mM, and (2) the concentrations of TMB was fixed at 1.6 mM. In detail, 100 μL of TMB (1–18 mM) and 10 μL of the pre-prepared His@AuNCs/GO (0.5 mg/mL) were added to 890 μL (pH 3.2, 50 mM) of acetate buffer and mixed thoroughly. After incubating at 25 °C for 5 min, the UV–visible spectrum was measured with a spectrophotometer.

We used the Lineweaver–Burk equation to calculate the kinetic parameters ($V_{\max}$ and $K_{m}$) [39]:

(1) $$v\;=\frac{V_{\max}\times \left[S\right]}{K_{m}+\left[S\right]},$$

where v is the reaction velocity, [S] is the concentration of the substrate, $V_{\max}$ is the maximum reaction velocity, and $K_{m}$ is the Michaelis–Menten constant [40].

### 2.5. Inhibiting of the Oxidase-Like Activity of His@AuNCs/GO by PPi

To check the inhibiting ability of PPi, 10 μL Na_4_P_2_O_7_·10H_2_O stock solution with different concentrations (0–6 mM) was added to 10 μL His@AuNCs/GO (0.5 mg/mL) and incubated at 25 °C for 10 min. The resultant solution was added to a solution containing 100 μL TMB (16 mM), and 880 μL of acetate buffer (pH 3.2, 50 mM) and stirred for 5 min at 25 °C. The UV–vis spectrum of the resultant solution was collected using a spectrophotometer.

### 2.6. Colorimetric Detection of ALP Activity

To determine the ALP activity, it is necessary to mix 10 μL of 4 mM PPi and freshly prepared ALP solutions of different concentrations, dilute to 100 μL with 0.05 M Tris-HCl buffer (pH 7.4), and incubate at 37 °C for 30 min (The incubation time was optimized to 30 min, Figure S4) [41]. Next, the above solutions and 10 μL His@AuNCs/GO (0.5 mg/mL) were mixed and incubated at room temperature for 10 min. The subsequent experimental steps were the same as those mentioned in Section 2.5 (final concentration of PPi: 40 μM). 

To evaluate the practical application of our proposed method, ALP in human serum samples was used for spiked experiments. Typically, human serum samples obtained from school hospital of Northwest normal university. The serum samples were diluted 50-fold with deionized water, and we performed the spike experiment with a known ALP concentration of 1 to 10 mU/mL.

## 3. Results

### 3.1. Characterization of His@AuNCs/GO

**Figure 1:**
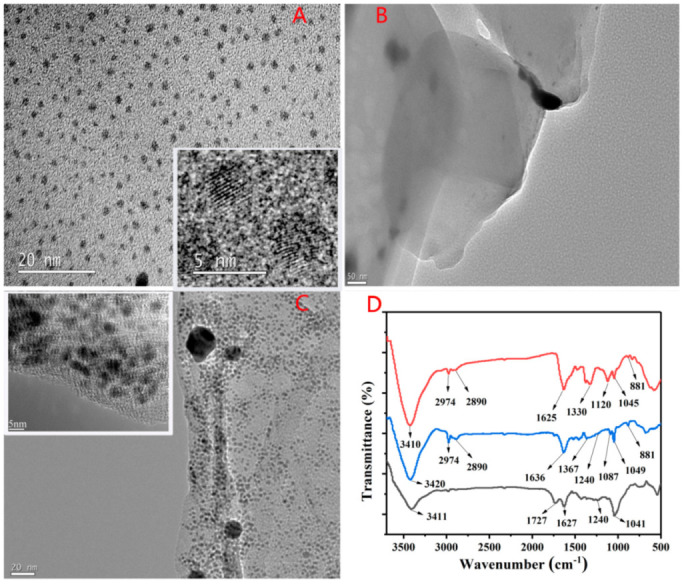
Characterization of His@AuNCs, GO, andHis@AuNCs/GO, respectively. (**A**) TEM of His@AuNCs, (**B**) TEM of GO, (**C**) TEM of His@AuNCs/GO, (**D**) FT-IR spectra of GO (black), His@AuNCs (red) and His@AuNCs/GO (blue). Insert in (A), (C) show HR-TEM of His@AuNCs and His@AuNCs/GO, respectively.

TEM was employed for the characterization of the morphology of His@AuNCs and His@AuNCs/GO. A typical TEM image (Figure 1A) showed that the His@AuNCs were highly uniform and monodisperse with a narrow size distribution of 1.72 nm (the size was statistically calculated from more than 100 NCs in the TEM image (Figure S5). In addition, the high-resolution transmission electron microscopy (HR-TEM) revealed a high crystallinity of the resulting His@AuNCs. 

As can be seen in Figure 1A (inset), the His@AuNCs showed clear lattice fringes with an interplanar distance of 0.24 nm, corresponding to the (111) lattice planes of the face-centered cubic structure of metallic Au (Figure 1A, inset). Figure 1B,C, respectively, shows the typical images before and after the loading of the His@AuNCs onto PDDA/GO. It may be observed (Figure 1C) that the GO nanosheets were modified by His@AuNCs. To further characterize, the FTIR spectra of GO, His@AuNCsand His@AuNCs/GO were record (Figure 1D). It can be observed that the characteristic peaks of GO (1041, 1240 cm^−1^) and His@AuNCs (881, 1330, 2974, 2890 cm^−1^) appear in His@AuNCs/GO at the same time [42,43]. Besides, Figure S6 shows EDX analysis of the GO, His@AuNCs, and His@AuNCs/GO, and the presence of the elements C, N, O, and Au were detected in His@AuNCs/GO. These results demonstrate that His@AuNCs effectively self-assembled on the PDDA/GO platform to form His@AuNCs/GO.

### 3.2. Oxidase-Like Activity of His@AuNCs/GO

**Figure 2:**
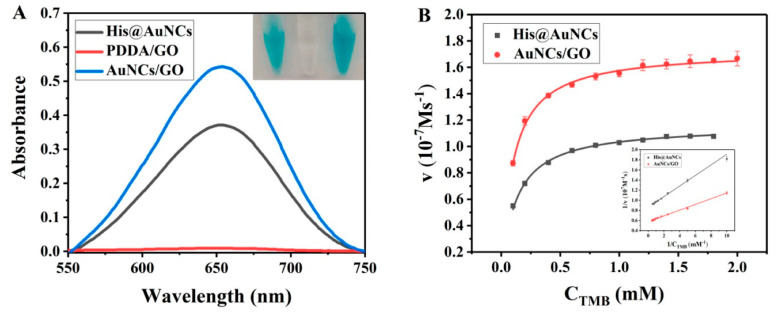
(**A**) The UV–vis absorption spectra of His@AuNCs, PDDA/GO, and His@AuNCs/GO in the colorimetric system with TMB as a substrate. The inset shows the corresponding photographs. Inset: photograph of corresponding solution (from left to right: His@AuNCs, PDDA/GO, and His@AuNCs/GO). (**B**) Michaelis–Menten curve of nanomaterials (His@AuNCs, His@AuNCs/GO) with different concentrations of TMB (inset: the Lineweaver–Burk plot of nanomaterials with different concentrations of TMB).

In our recent work, the oxidase mimic activity of His@AuNCs was studied by the catalytic oxidation of the substrate TMB [37]. In this work, the oxidase-like activity of the His@AuNCs loaded onto PDDA/GO was investigated. As shown in Figure 2A, compared with the His@AuNCs-TMB system, a visible color change was observed in the His@AuNCs/GO-TMB system (inset of Figure 2A). This result was also evidenced by the UV–vis absorption spectrum at 652 nm. The results indicated that His@AuNCs/GO has an oxidase-like activity. We further studied the catalytic properties of His@AuNCs/GO through steady-state kinetic experiments. 

As seen in Figure 2B, the initial rate versus TMB concentration followed typical Michaelis–Menten behaviors for a certain range of substrate concentrations. Under the same conditions, the absorption of the His@AuNCs-TMB solution was further investigated. The $K_{m}$ and the $V_{\max}$ were calculated using the Lineweaver–Burk plot [37]: the $K_{m}$ and $V_{\max}$ of His@AuNCs/GO were found to be 0.098 mM and 1.728 × 10^−7^ M s^−1^, respectively, and those of His@AuNCs were found to be 0.126 mM and 1.164 × 10^−7^ Ms^−1^, respectively (Table 1). The results indicated that His@AuNCs/GO in comparison with His@AuNCs had a higher affinity toward TMB, that is, a better oxidase-like catalytic activity.

### 3.3. Inhibitory Effects of PPi

**Figure 3:**
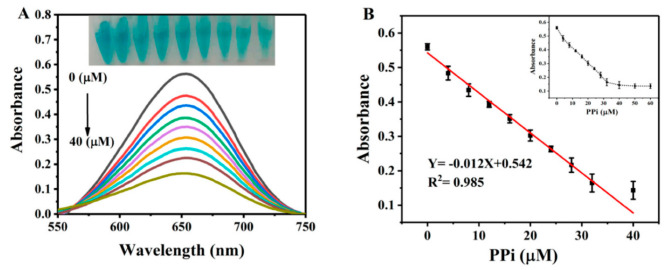
(**A**) The UV–vis absorption spectra of the His@AuNCs/GO-TMB system in the presence of various concentrations of PPi. Inset shows the photos of the corresponding solutions. (**B**) Linear relationship for PPi detection. Absorbance changes with the increase of PPi concentration from 4 to 60 μM (inset). Error bars represent the standard deviations (*n* = 3).

As the proposed method was based on the modulation of the oxidase-like activity of His@AuNCs/GO, we first investigated the inhibitory effects of PPi on the activities of His@AuNCs/GO. Different amounts of PPi and His@AuNCs/GO were mixed and added into the catalytic oxidation reaction solution containing TMB. The typical absorption spectra around 652 nm are shown in Figure 3A. Figure 3B shows the dose-related inhibitory ability of PPi on the oxidase-like activity of His@AuNCs/GO; the absorption signals were linear with the PPi observed within the range of 0–40 μM (Y=−0.012X+0.542, R^2^ = 0.985). As the maximum inhibition of the catalytic activity occurred when 40 μM of PPi was added, the concentration of 40 μM was selected for subsequent detection. Moreover, the inhibitory effects of PPi to the oxidase-like activity of His@AuNCs were investigated. As shown in Figure S7, the inhibitory effects of PPi to the His@AuNCs are far lower than that to His@AuNCs/GO, indicating that His@AuNCs/GO is needed in the proposed method.

According to the previous reports about nanozymes, in the presence of reducing agents, TMB is less effectively oxidized, which may be due to different mechanisms—namely, reducing oxTMB to colorless TMB [44,45,46]. To clearly explain the inhibitory behavior of PPi, we explored whether PPi can reduce oxTMB. Figure S8 shows the absorbance at 652 nm when PPi is added to the His@AuNCs/GO-TMB system after 5 min of chromogenic reaction. Compared with the system without PPi, there was no obvious change in absorbance, which proves that PPi cannot reduce oxTMB. Therefore, we suggest that the inhibition of PPi to His@AuNCs/GO is similar to previous reports [47,48], as shown in Scheme 1, as PPi has strong affinities toward His@AuNCs/GO [49,50].

### 3.4. Detection of ALP Activity

**Figure 4:**
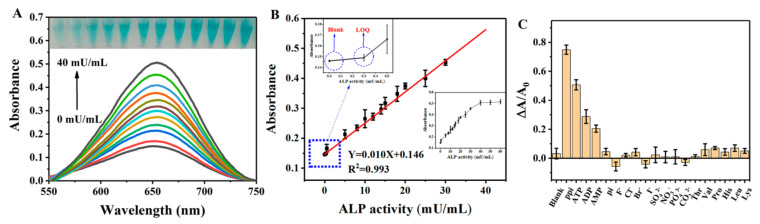
(**A**) The UV–vis absorption spectra of the His@AuNCs/GO-TMB system in the absence and presence of various concentrations of ALP; the inset shows the photos of corresponding solutions. (**B**) Linear relationship between the absorbance at 652 nm and the ALP concentration. Absorbance changes with the increase of ALP concentration from 0.5 to 60 mU/mL (inset). Error bars represent the standard deviations (*n* = 3) (**C**) Selectivity of the assay for PPi compared to other potential interferences. Experimental conditions: PPi (40 μM), other anions (200 μM), and amino acids (500 μM). Error bars represent the standard deviations (*n* = 3).

ALP can catalyze the hydrolysis of PPi in a weak medium to generate Pi. Thus, we hypothesize that it is possible to restore the inhibited activity of nanozymes when ALP is incubated with PPi/His@AuNCs/GO (His@AuNCs/GO treated with PPi) via the hydrolysis of PPi. With this assumption, we designed a colorimetric ALP activity assay (Scheme 1), and evaluated the sensitivity of the colorimetric analysis in the detection of the ALP activity. Figure 4A shows the absorption spectra of the ALP-treated PPi/His@AuNCs/GO-TMB system with different concentrations of ALP. The absorption signal increased with increasing ALP concentration. 

To verify whether Pi and ALP are directly involved in the restoration of the inhibited catalytic activity of His@AuNCs/GO, we performed control experiments by adding Pi and ALP to the His@AuNCs/GO-TMB system. As seen in Figure S9, no color change was seen in the control tests. The results confirmed the feasibility of the proposed strategy for ALP activity sensing (Scheme 1). Under optimal conditions, the absorbance values of ALP solutions with different ALP concentrations were measured; the absorption signals were linear with the ALP activities observed within the range of 0–40 mU/mL (Figure 4B) (Y=0.010X+0.146, R^2^ = 0.993) and the limit of detection (LOD) for ALP was 0.26 mU/mL evaluated using a signal three-fold the background noise (S/N = 3), and a LOQ of 0.30 mU/mL at three times background noise is measured. The ALP activity detection established by this method was better than previously reported, as listed in Table 2. 

The assay method we proposed can distinguish the color change caused by ALP with the naked eye, is easy to implement, and meets the sensitivity requirements, and this method does not require expensive instruments to detect ALP [47]. To evaluate the selectivity of the proposed method, an investigation on the influence of potentially interfering substances, including ATP, ADP, AMP, Pi, F^−^, Cl^−^, Br^−^, I^−^, SO_4_^2−^, NO^3−^, PO_4_^3−^, CO_3_^2−^, Thr, Val, Pro, His, Leu, and Lys, was performed under the optimized conditions. We observed that the abovementioned coexisting interfering substances had a negligible effect on the absorption intensity (Figure 4C). The results demonstrate that, due to the strong coordination interaction between PPi and His@AuNCs/GO and the specific catalytic activity of ALP on PPi, the selectivity of the sensor was satisfactory.

The robustness of the proposed method was evaluated by the relative standard deviation (RSD) of sensing affect the His@AuNCs/GO to ALP activity. The results of detection from His@AuNCs/GO of different synthetic batches under the same conditions were shown in Figure S10A. The calculated RSD were 2.57%, indicating the proposed method has good accuracy. In addition, the stability of His@AuNCs/GO was evaluated. The His@AuNCs/GO was stored at 4 °C for one month; afterwards, 95% of the oxidase-like activity of His@AuNCs/GO was still preserved compared with the initial value, and the sensing properties of His@AuNCs/GO to ALP did not change significantly (Figure S10B). These results indicated that the proposed method have good repeatability and outstanding stability.

### 3.5. Analytical Application in Real Samples

To assess the potential applicability of the method, we conducted an assay of the ALP activity in serum samples with different ALP concentrations. As shown in Table 3, the recovery efficiency and RSD values were obtained in the range of 98.67–102.5% and 2.08–4.44%, respectively, for different samples, indicating the effectiveness and reliability in the application of the proposed His@AuNCs/GO hybrid-based colorimetric assay in real biological samples.

## 4. Conclusions

In summary, we proposed a facile colorimetric assay for the sensitive and selective determination of the ALP activity based on the PPi-inhibited oxidase mimic activity of His@AuNCs/GO. The hydrolysis of PPi by ALP restored the inhibited activity of His@AuNCs/GO, enabling His@AuNCs/GO to efficiently catalyze the oxidation of TMB to generate the blue product oxTMB. The intensity of the color showed a linear dependency (0–40 mU/mL) and can be related to the ALP activity. This is a fast, robust, simple, and cost-effective method that can be used to accurately and reliably detect ALP activity in real samples, which suggests that it has good practicability and reliability in detecting ALP activity in clinical practice.

## Figures and Tables

**Table 1:** Apparent steady-state kinetics parameter for nanomaterials oxidase mimic activity.

Catalyst	$K_{m}\;(\mathrm{mM})$	$V_{\max}$ (10^−7^ M s^−1^)
His@AuNCs (TMB)	0.126	1.164
His@AuNCs/GO (TMB)	0.098	1.728

**Table 2:** Comparison of our method with other ALP detection methods.

Material	Method	Linear Range (mU/mL)	LOD (mU/mL)	References
Au NPs/ATP	Colorimetric	100–600	10	[51]
Au nanorods	Colorimetric	5–100	3.3	[14]
CdTe/CdS QDs	Fluorescent	3–1000	3	[52]
CDs	Fluorescent	2.5–40	1	[53]
GQDs	Fluorescent	16.7–782.6	1.1	[54]
Protein stabilized gold nanocubes	Fluorescent	0.0312–1	1.616	[55]
PDA nanodots	Colorimetric	1–50	0.94	[56]
His@AuNCs/GO	Colorimetric	0–40	0.26	This work

**Table 3:** Reliability of His@AuNCs/GO for ALP activity detection in real samples (*n* = 3).

Sample	Added (mU/mL)	Detected (mU/mL)	RSD (%, *n* = 3)	Recovery (%)
1	3	2.96	3.22	98.67
2	4	3.97	4.44	99.17
3	6	6.15	2.08	102.50
4	9	9.15	2.24	101.70

## Data Availability

The data presented in this study are available on request from the corresponding author.

## Supplementary Materials

The following are available online at https://www.mdpi.com/article/10.3390/bios11060174/s1, Electronic supplementary information (ESI) available. Figure S1: UV-Vis absorption spectra of His@AuNCs/GO-TMB system with different incubation ratios of His@AuNCs and PDDA-GO (from a to f: 1:10, 1:20, 1:30, 1:40, 1:50, 1:100), Figure S2: UV-Vis absorption spectra of His@AuNCs/GO-TMB system in different concentrations of His@AuNCs/GO (from a to e: 0.002, 0.003, 0.004, 0.005, 0.006 mg/mL), Figure S3: Effect of pH (a), acetate buffer (b) and TMB concentration on the oxidase-like activity of His@AuNCs/GO, Figure S4: The absorbance of the sensing system as a function of the enzymatic reaction time (0, 5, 10, 15, 20, 25, 30, 40, 60 min) in the presence of 0, 10, and 20 mU/mL ALP individually, with PPi as the substrate, Figure S5: The size statistical histograms of His@AuNCs, Figure S6: EDX of His@AuNCs (A), GO (B) and His@AuNCs/GO (C), Figure S7: The absorbance of the sensing His@AuNCs-TMB system with PPi as the substrate, The inset is the inhibitory effect of PPi on His@AuNCs/GO-TMB system, Figure S8: Comparison of the UV-Vis absorption spectra of the His@AuNCs/GO-TMB system after 5 min of reaction with PPi and without PPi, Figure S9: UV-vis absorption spectra of His@AuNCs/GO-TMB system in the presence of ALP and Pi, Figure S10 (A): Detection of ALP activity by colorimetric sensor established by different batches of His@AuNCs/GO, (B): a represents the oxidase-like activity of His@AuNCs/GO, b and c respectively represent the detection of PPi and ALP by the sensor.
