# Supplementary material for: Restoring the Oxidase-Like Activity of His@AuNCs for the Determination of Alkaline Phosphatase
Source: Biosensors (Basel). 2021 May 30;11(6):174. doi: 10.3390/bios11060174 (PMC8227771; doi:10.3390/bios11060174)
Supplement: Supplementary file 1 [file biosensors-11-00174-s001.zip › biosensors-1189625-sp.pdf]

biosensors

Supplementary Materials

Restoring the Oxidase-Like Activity of His@AuNCs for the Determination of Alkaline Phosphatase

Fanfan Xiao [1,†], Yuting Yu [1,†], Yang Wu [1], Lili Tian [1], Guoyan Zhao [1], Hailong Pang [1] and Jie Du [1,2,*]

[1] College of Life Sciences, Northwest Normal University, 967 Anning Road, Lanzhou 730070, China; 2018212256@nwnu.edu.cn (F.X.); 2017212141@nwnu.edu.cn (Y.Y.); 2020212651@nwnu.edu.cn (Y.W.); 2018212262@nwnu.edu.cn (L.T.); 2018212254@nwnu.edu.cn (G.Z.); Phl-3088@163.com (H.P.)

[2] Key Laboratory of Bioelectrochemistry & Environmental Analysis of Gansu Province, Northwest Normal University, Lanzhou 730070, China

* Correspondence: dujie@nwnu.edu.cn

† These authors have made equal contributions to this work and should be considered as co-first authors.

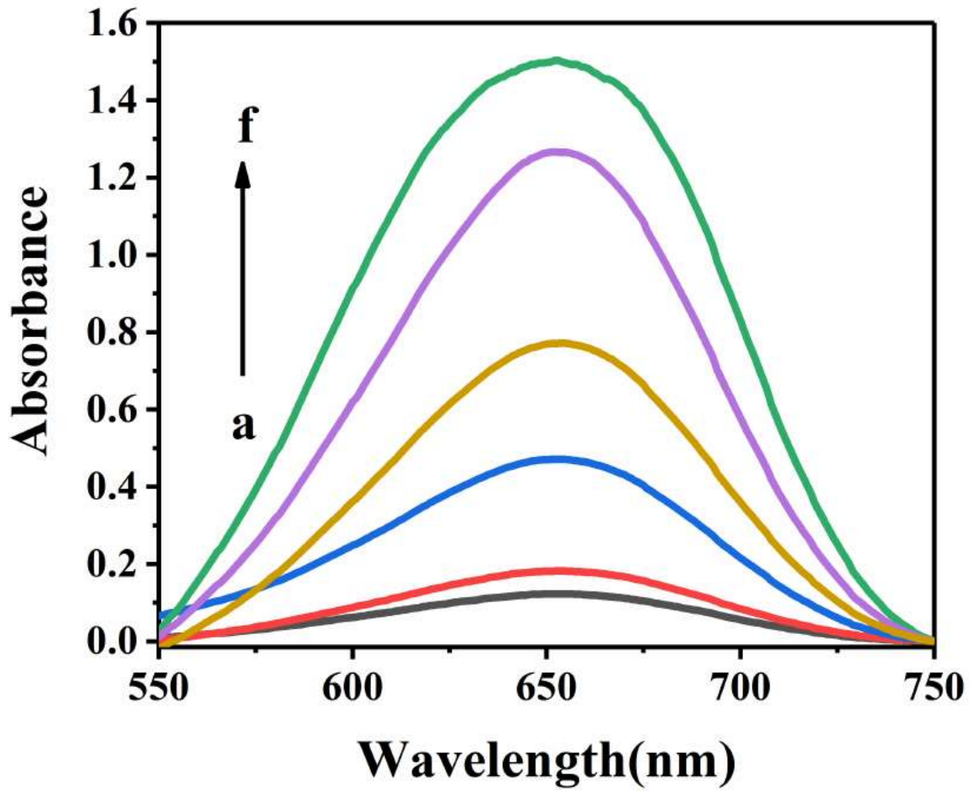

Figure S1. UV-Vis absorption spectra of His@AuNCs/GO-TMB system with different incubation ratios of His@AuNCs and PDDA-GO (from a to f: 1:10,1:20,1:30,1:40,1:50,1:100).

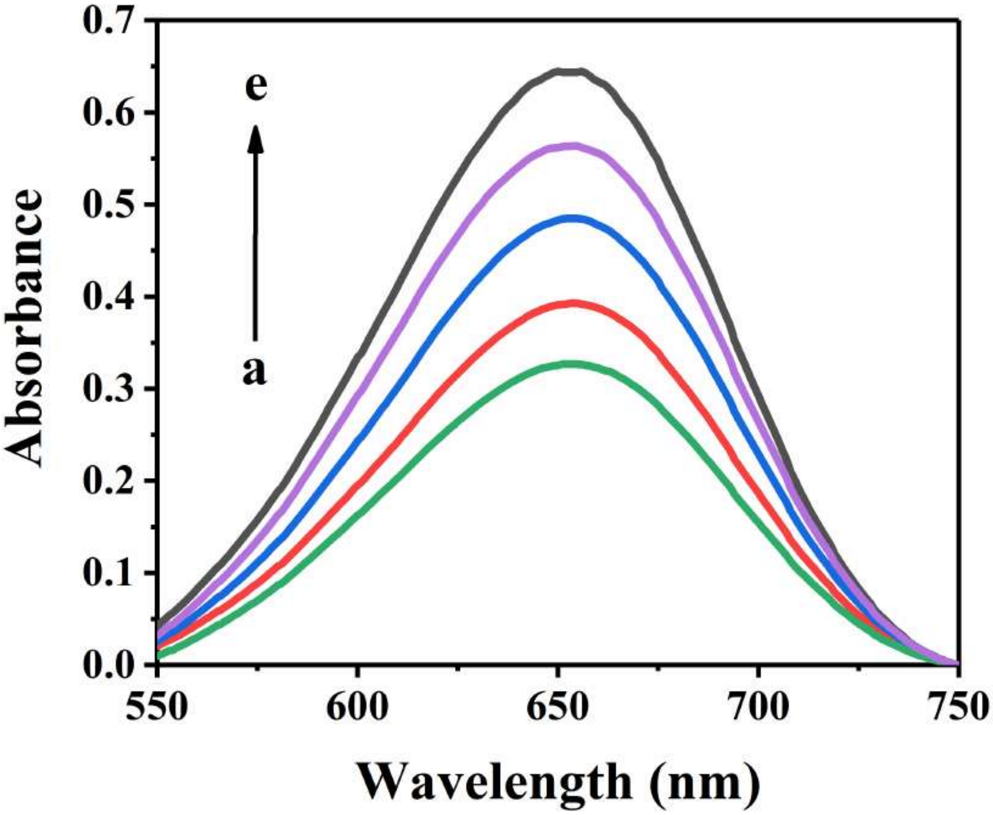

Figure S2. UV-Vis absorption spectra of His@AuNCs/GO-TMB system in different concentrations of His@AuNCs/GO (from a to e:0.002,0.003,0.004,0.005,0.006mg/mL).

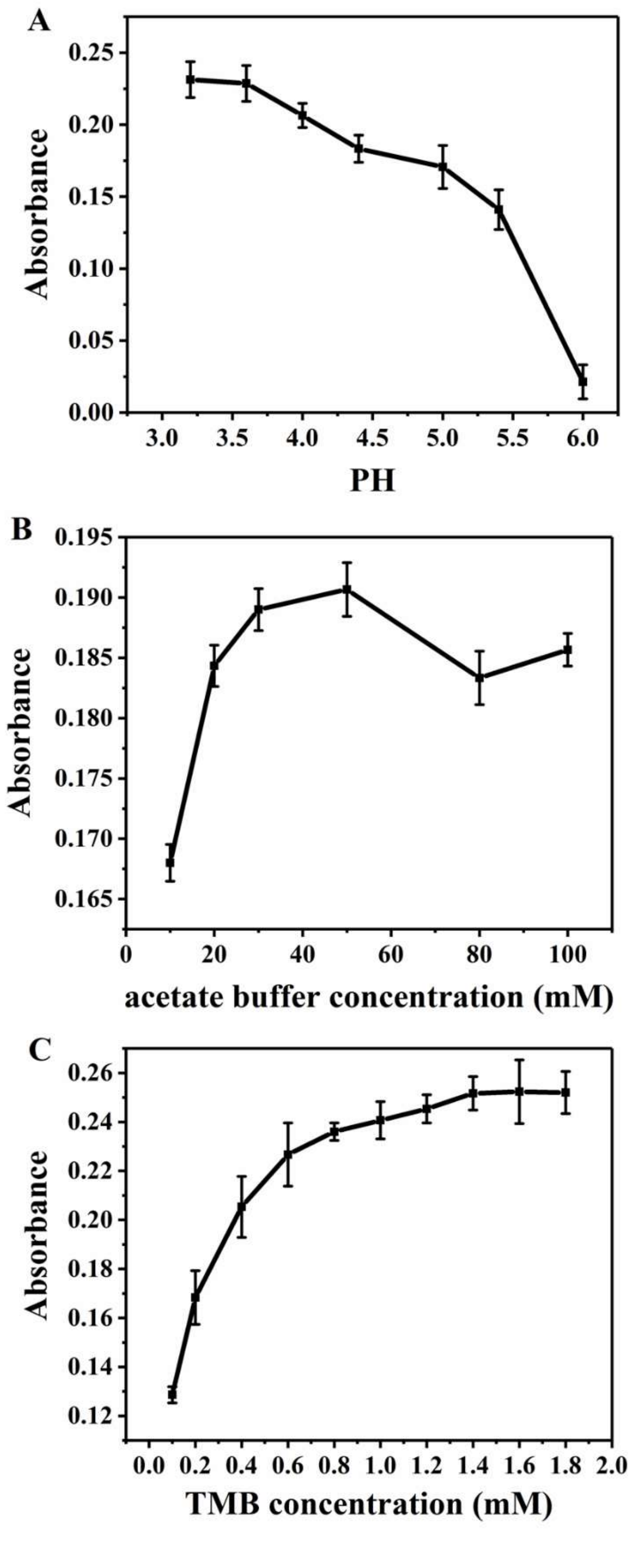

Figure S3. Effect of pH (a), acetate buffer (b) and TMB concentration on the oxidase-like activity of His@AuNCs/GO.

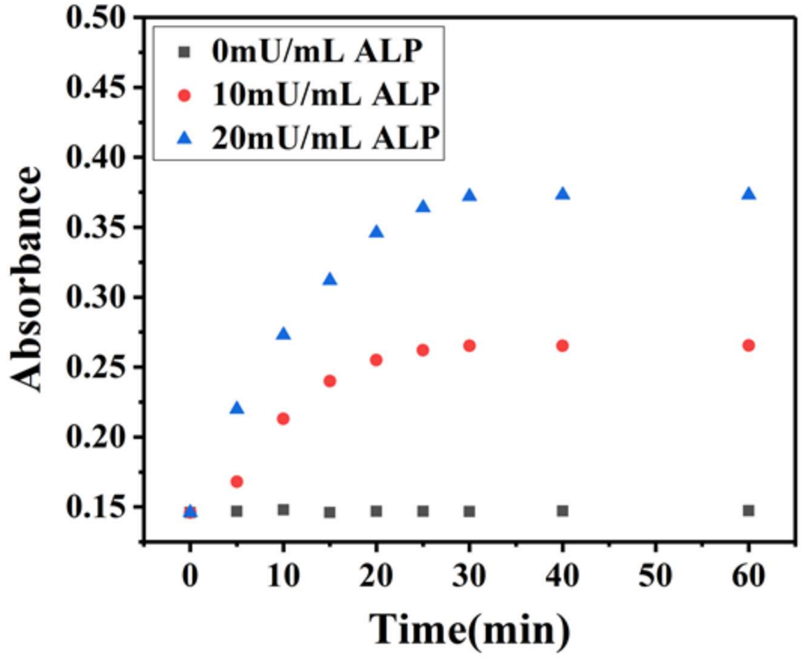

Figure S4. The absorbance of the sensing system as a function of the enzymatic reaction time (0, 5, 10, 15, 20, 25, 30, 40, 60 min) in the presence of 0, 10, and 20 mU/mL ALP individually, with PPi as the substrate.

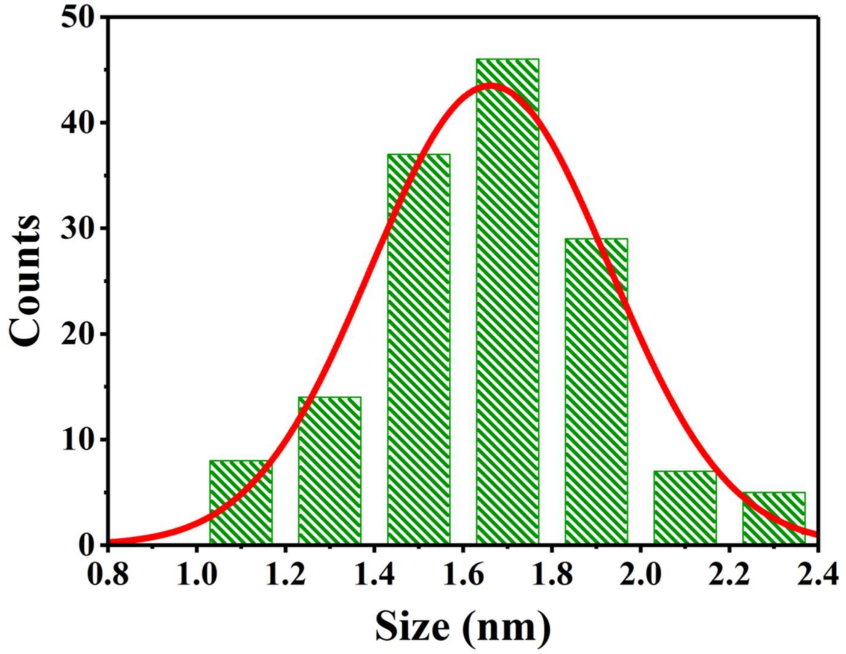

Figure S5. The size statistical histograms of His@AuNCs.

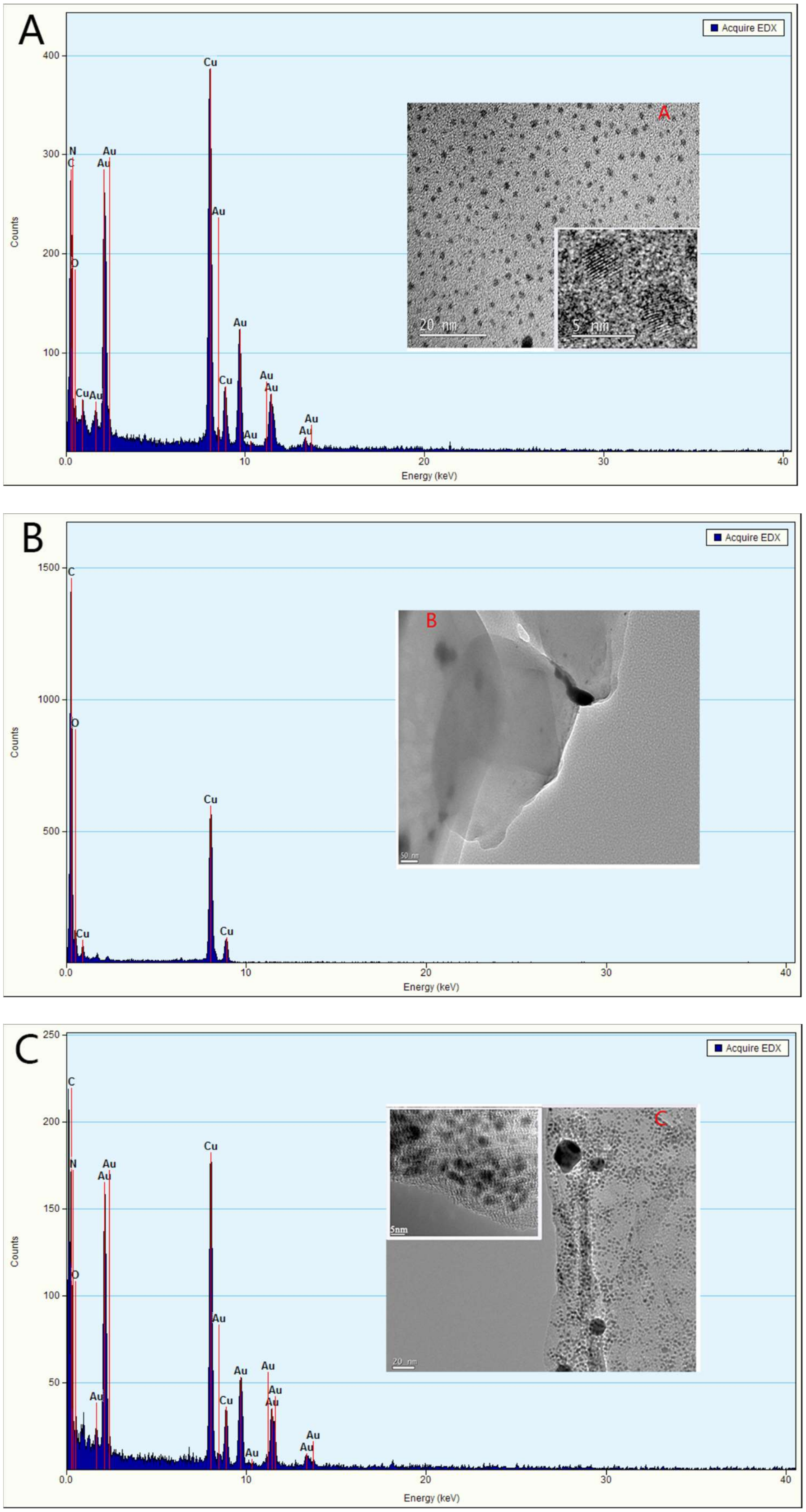

Figure S6. EDX of His@AuNCs (**A**) ,GO (**B**) and His@AuNCs/GO (**C**).

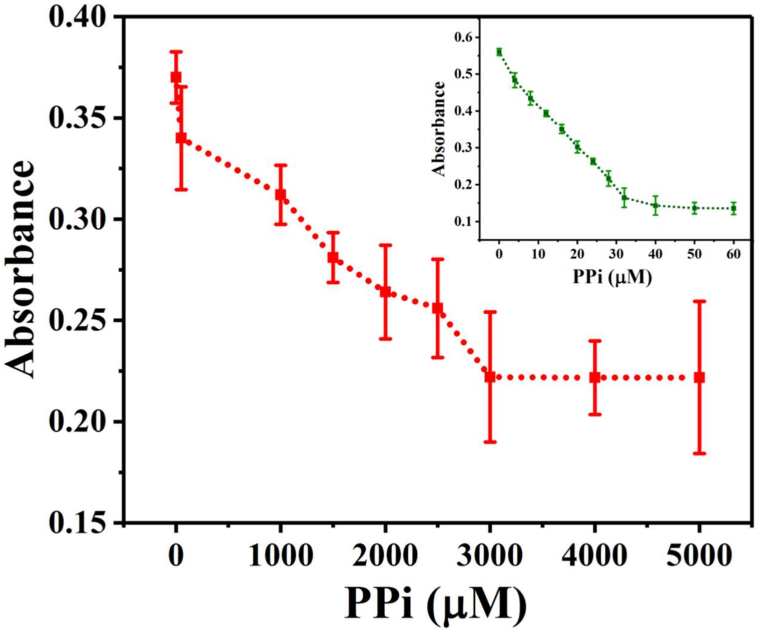

Figure S7. The absorbance of the sensing His@AuNCs-TMB system with PPi as the substrate. Inset is the inhibitory effect of PPi on His@AuNCs/GO-TMB system.

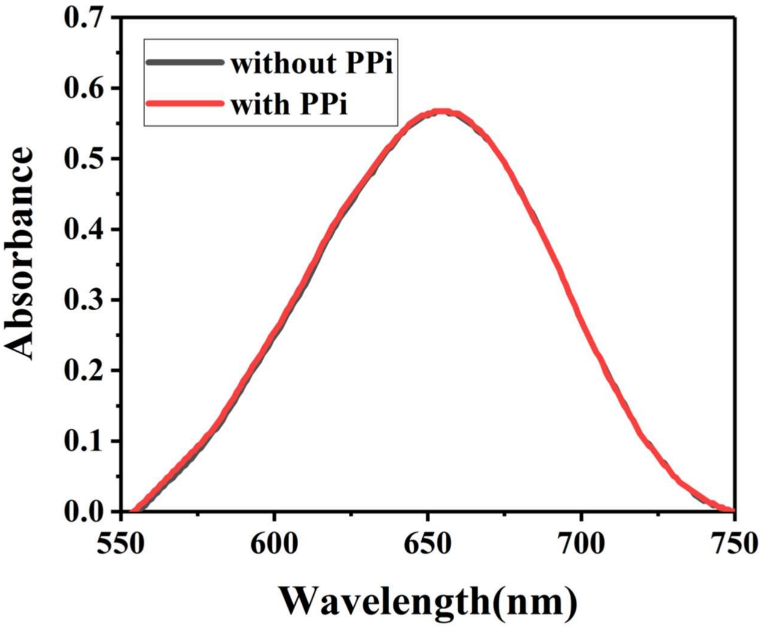

Figure S8. Comparison of the UV-Vis absorption spectra of the His@AuNCs/GO-TMB system after 5 min of reaction with PPi and without PPi.

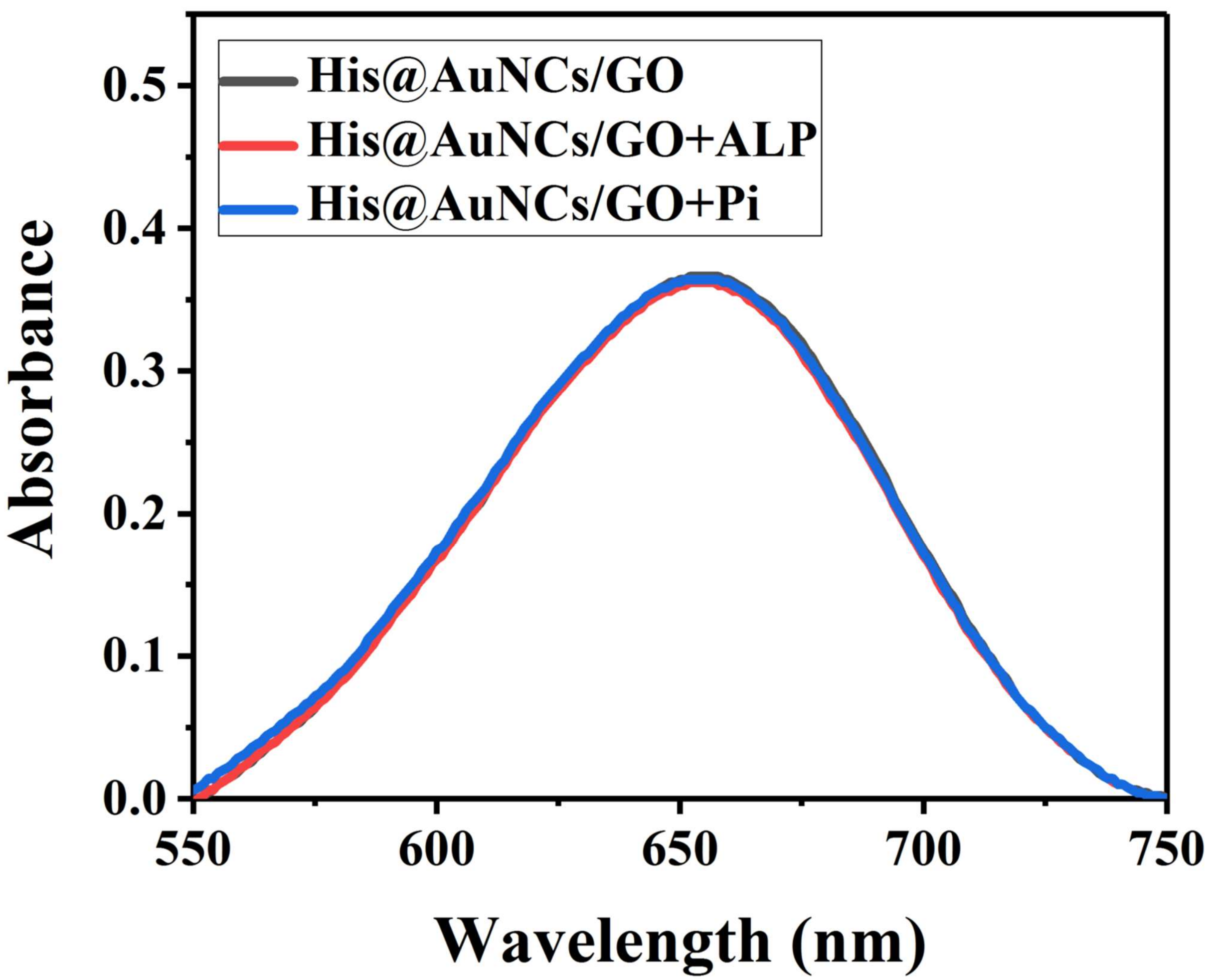

Figure S9. UV-vis absorption spectra of His@AuNCs/GO-TMB system in the presence of ALP and Pi.

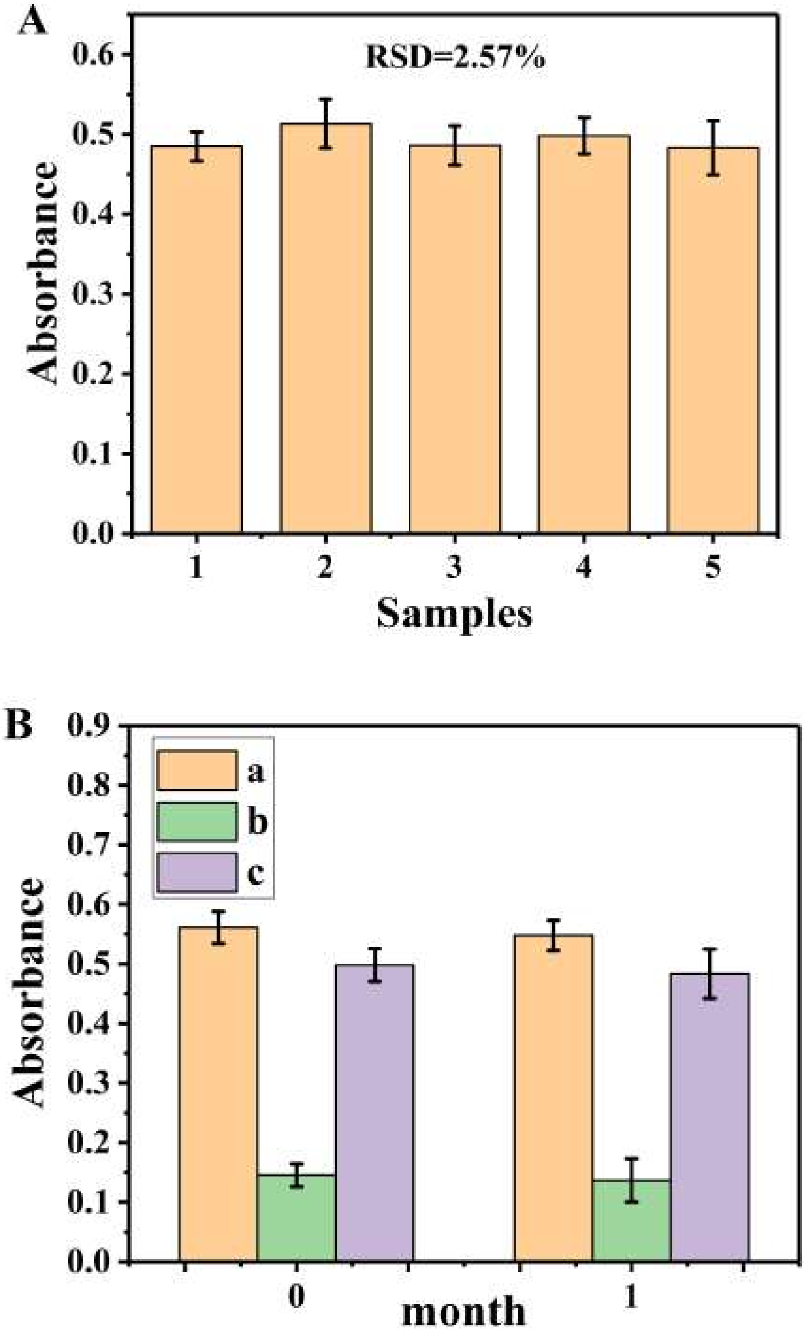

Figure S10. (**A**) Detection of ALP activity by colorimetric sensor established by different batches of His@AuNCs/GO. (**B**) a represents the oxidase-like activity of His@AuNCs/GO, b and c respectively represent the detection of PPi and ALP by the sensor.
